# Mechanisms of interaction between type 2 diabetes and psychological disorders and therapeutic interventions: a narrative review

**DOI:** 10.3389/fmed.2026.1777797

**Published:** 2026-04-29

**Authors:** Haoxuan Sui, Yujie Sun, Haoming Yu, Shengyu Han, Jihong Li, Lei Wan, Yuhua Chi

**Affiliations:** 1 Department of General Medicine, Affiliated Hospital of Shandong Second Medical University, Weifang, Shandong, China; 2 College of Clinical Medicine, Shandong Second Medical University, Weifang, Shandong, China

**Keywords:** gut microbiota, intervention measures, mechanism of action, psychological disorders, type 2 diabetes

## Abstract

Diabetes mellitus is a metabolic disorder characterised by hyperglycaemia, having become one of the most prevalent diseases globally. Type 2 diabetes constitutes the most common form amongst diabetic patients. As the condition progresses, patients typically require long-term medication, imposing significant burdens on daily life. Prolonged treatment is liable to trigger psychological problems such as anxiety, depression and cognitive impairment, which exerts an adverse impact on the long-term management and prognosis of the condition. In recent years, the association between the gut microbiota and diabetes has been increasingly recognised, with the microbiota-gut-brain axis (MGBA) playing a significant role. Consequently, this review primarily explores the role of gut microbiota in psychological disorders amongst patients with type 2 diabetes (T2DM), briefly outlines the association between T2DM and psychological disorders. This article also discusses various therapeutic interventions aimed at alleviating psychological symptoms in patients with Type 2 Diabetes Mellitus (T2DM), including pharmacological treatments such as GLP-1 receptor agonists (GLP-1RA) and DPP-4 inhibitors, which not only show potential in regulating blood glucose but also have beneficial effects on mood health. Furthermore, we emphasise lifestyle interventions as an adjunctive approach, which provides a positive impact on improving psychological well-being. This review comprehensively outlines the mechanisms linking T2DM and psychological disorders, offering clinicians a thorough overview of the subject and valuable reference material.

## 1 Introduction

Type 2 diabetes mellitus (T2DM) is the most prevalent chronic metabolic disorder, occurring widely across the globe. Reports indicate that approximately 529 million people worldwide had diabetes in 2021, with projections suggesting this figure will rise to around 1.31 billion by 2050, making it one of the most common chronic metabolic diseases globally (1). The primary core mechanisms are insulin resistance and impaired insulin secretion (2), arising from the interaction of genetic and environmental factors. Psychological stress and sedentary lifestyles further exacerbate these factors (3). Gut microbiota dysbiosis is associated with altered intestinal permeability and is implicated in T2DM (4, 5), involving interactions between chronic inflammation, immune dysregulation, and metabolism (6). The microbiota-gut-brain axis (MGBA) facilitates intimate crosstalk between the gut and the brain via diverse pathways, including microbial, neural, endocrine, metabolic and immune signalling, thereby exerting an important communicative role (7).

Type 2 diabetes mellitus not only impacts physical health but is also associated with a high prevalence of psychological disorders, particularly anxiety and depression (8). It is further complicated by cognitive disturbances, with the intricate relationship between these conditions being mediated by the gut-brain axis. In patients with psychological disorders, beneficial gut microbiota such as Lactobacillus, Bifidobacterium, and Faecalibacterium diminish, while potentially harmful bacteria proliferate, contributing to dysbiosis (9). This disruption of the gut microbiome is associated with patients’ emotional states through neural, metabolic, and inflammatory pathways (10). Gut microbiota may interact with the brain through direct or indirect pathways, such as short-chain fatty acids, cytokines, serotonin, and gamma-aminobutyric acid, thereby altering the integrity of the blood–brain barrier. This can subsequently influence brain function and exacerbate the development of psychological disorders (11). Concurrently, mental health issues can adversely affect patients’ glycaemic control, leading to a significant decline in quality of life and self-care capabilities, which substantially impedes the therapeutic process (12). This paper provides an overview of the role of gut microbiota in psychological disorders amongst patients with T2DM, briefly outlines the association between T2DM and psychological disorders, and subsequently explores therapeutic interventions for patients with T2DM. It aims to furnish clinicians with comprehensive information and act as a reference for clinical practise.

Individuals with T2DM exhibit distinct mechanisms within the microbiota-gut-brain axis compared with those affected by obesity and metabolic syndrome. Chronic hyperglycaemia in T2DM not only contributes to the dysbiosis of the gut microbiota but also impairs intestinal barrier function, allowing harmful substances from the gut to enter the systemic circulation and reach the brain, thereby contributing to the development of psychological disturbances such as depression and anxiety.

Furthermore, persistent insulin resistance present in T2DM leads to impaired insulin signalling, which disrupts the normal transmission of neurotransmitters to the brain and interferes with emotion-related neural pathways, predisposing individuals to adverse mood states. In comparison, although individuals with obesity similarly exhibit insulin resistance and may present with mild inflammatory responses, their blood glucose levels do not typically rise persistently and markedly to the same extent as those with T2DM. Consequently, the chronic hyperglycaemic neurotoxicity commonly observed in patients with T2DM is generally absent in this population. This renders T2DM a distinct clinical population for investigating the impact of hyperglycaemia on mental health via the microbiota-gut-brain axis.

A systematic literature search strategy was employed in this review. Relevant databases including PubMed and CNKI were searched from 2017 to 2025 to help ensure coverage of the most recent research findings. The primary search terms included “type 2 diabetes mellitus,” “psychological disorders,” “anxiety,” “depression,” “gut microbiota,” and “microbiota-gut-brain axis (MGBA).” Studies were considered eligible for inclusion if they enrolled patients with type 2 diabetes mellitus, investigated anxiety, depression or other related psychological disorders, and adopted a study design of clinical research, experimental study or systematic review. Studies that did not explore the interrelationship between type 2 diabetes mellitus and psychological disorders were excluded. Following screening, a total of 95 eligible studies were ultimately included for analysis and discussion. Implementation of this approach helped support the comprehensiveness and reliability of the literature search, providing a robust evidence base for this narrative review.

## 2 Interaction mechanisms between T2DM and psychological disorders

### 2.1 Depression

Individuals with T2DM require long-term medication management. Financial and life pressures may lead to low mood, diminished interest, self-blame, or even pessimism and hopelessness, making them susceptible to depressive states. Depression affects one-third of diabetes patients globally and correlates with age, gender, social circumstances, and clinical factors. Research indicates that T2DM patients under 60 years of age, women (13), smokers, drinkers, those with poor social support networks, high stress levels and existing cerebral microvascular complications face a heightened risk of developing depression (14, 15). Furthermore, a number of studies indicate that medication use (including metformin, insulin, antidepressant), obesity dietary patterns, physical inactivity, inflammatory responses, and socioeconomic stress may be significantly associated with the development of depression in patients with T2DM (16). Specifically, adverse drug effects, chronic inflammation secondary to obesity, poor nutritional intake, insufficient physical activity, and low socioeconomic status appear to potentially exacerbate depressive symptomatology. Epidemiological data demonstrate an elevated prevalence of depression amongst T2DM patients, while individuals with depression also face an increased risk of developing diabetes, with the two conditions frequently coexisting (17). The American Diabetes Association (ADA) guidelines emphasise that depression significantly impairs diabetes self-management and quality of life, adversely affecting prognosis (18). T2DM is associated with excessive activation of mechanisms including chronic inflammation, insulin resistance, the hypothalamic–pituitary–adrenal (HPA) axis, and the microbiota-gut-brain axis, which is linked to the development of depressive states. The physiological responses of diabetic patients involve elevated levels of stress-related hormones, such as cortisol (19), which can increase pro-inflammatory cytokines like tumour necrosis factor-*α* (TNF-α) and interleukin-6(IL-6). Concurrently, in patients with T2DM, persistent hypercortisolaemia and chronic stress may blunt negative feedback, potentially leading to excessive hypothalamic secretion of CRH and pituitary ACTH. This may further stimulate cortisol production, causing aberrant HPA axis activation and potentially exacerbating disease progression (20). The HPA axis is a brain region that controls stress responses and regulates various bodily processes (21);its dysregulation may increase the risk of depressive complications. The majority of neurotransmitters present in the human brain, such as serotonin, gamma-aminobutyric acid, and short-chain fatty acids, are associated with the metabolic activity of the gut microbiota (22). Hyperactivation of the HPA axis, inducing hyperglycaemia and hypercortisolemia, is associated with disruptions to the gut microbiota. This disruption causes dysbiosis and impaired intestinal barrier function, allowing increased levels of inflammatory bacterial products such as lipopolysaccharides (LPS) to enter the bloodstream and ultimately reach the brain, which is linked to the development of depressive states.

T2DM is also associated with increased intestinal permeability, potentially inducing LPS-mediated metabolic endotoxinaemia. This damages *β*-cells and activates TLR4 on microglia, while also increasing pro-inflammatory cytokine release, thereby exacerbating depressive symptoms (23). Conversely, depression can stimulate the HPA axis, elevating cortisol and inflammatory cytokine levels, thereby exacerbating glycaemic fluctuations and contributing to diabetes onset (24). Furthermore, depression may be associated with gut microbiota dysbiosis, which can affect patient health through metabolic pathways involving short-chain fatty acids and tryptophan, thereby increasing diabetes risk (25).

### 2.2 Anxiety

Factors such as prolonged poor glycaemic control, reduced adherence to self-care, and increased incidence of complications may all contribute to the development of anxiety. This typically manifests as persistent states of tension characterised by restlessness and excessive worry. Anxiety is further influenced by gender, age, psychosocial stressors, and associated complications. Its pathogenesis is primarily linked to the basolateral amygdala complex and hippocampal regions involved in learning processes (26, 27). Studies have suggested that hippocampal neuronal apoptosis and synaptic structural damage may lead to excessive activation of microglia and astrocytes (28), resulting in the overexpression of pro-inflammatory cytokines including TNF-*β* and IL-6. This may subsequently contribute to insufficient secretion of dopamine and 5-hydroxytryptamine, as well as elevated norepinephrine levels, which could further promote the development of anxiety-related behaviours and symptoms. However, the TREM2 receptor present on microglia plays a crucial role in synapse protection, regulating cellular phenotype switching, and anti-inflammatory responses (29). Triggering Receptor Expressed on Myeloid Cells 2 (TREM2) is a receptor expressed on the surface of microglia, and is widely distributed in immune cells within the central nervous system. This receptor is thought to play a crucial role in modulating immune responses, preserving synaptic function, and maintaining homeostatic stability within the nervous system.

Pro-inflammatory cytokines released by overactivated microglia indirectly act upon the TREM2 receptor, inducing its upregulation. This elevated TREM2 then inhibits the IκB-NADPH oxidase-receptor activator of nuclear factor κB-STAT pathway. This dual mechanism promotes increased release of anti-inflammatory factors such as IL-10 by the microglia themselves, while simultaneously enhancing synaptic activity. Consequently, this alleviates the patient’s state of anxiety (30). Collaboration with basic medical research may be strengthened to provide support for clinical work through fundamental studies on the TREM2 receptor.

Increasing research also indicates that gut microbiota is associated with anxiety states (7). The gut microbiome produces neurotransmitters such as gamma-aminobutyric acid and serotonin, substances intrinsically linked to anxiety (31). Consistent with depressive mechanisms, T2DM can activate the HPA axis, inducing intestinal immune activation and disruption of the gut microbiota. This results in increased inflammatory bacterial factors crossing the blood–brain barrier, excessively activating microglia, thereby exacerbating neuroinflammation and ultimately inducing anxiety in patients. Moreover, T2DM interacts with anxiety. Anxiety also activates the HPA axis, inducing increased release of stress hormones such as cortisol and adrenaline. This impairs insulin sensitivity, thereby disrupting the endocrine system and causing blood glucose dysregulation, resulting in a hyperglycaemic state (32). Depression and anxiety have become common mental disorders arising from alterations in cognition, emotion, and attitude, significantly impacting individuals’ health and daily lives (33, 34). Amongst patients with T2DM and co-occurring psychological disorders, depression and anxiety represent the most prevalent mental health conditions. A recent meta-analysis demonstrated that the pooled prevalence of comorbid depression in individuals with T2DM was 19% (35), and the prevalence of anxiety disorders was 13.7% (36). These prevalence estimates were significantly elevated compared with non-diabetic control populations. A stable and statistically significant disease-specific association was observed between T2DM and both depression and anxiety. Subsequent analysis indicated that T2DM was associated with an increased risk of incident depression and anxiety, and may function as an important independent risk factor for these mental health disorders.

### 2.3 Cognitive impairment

Cognitive impairment constitutes a brain disorder associated with diabetes mellitus, characterised by distinctive structural abnormalities in the brain alongside diminished learning and memory capabilities (37). Primary manifestations include impaired memory function, difficulties in task execution, attention deficits, and impaired verbal fluency. The American Diabetes Association guidelines explicitly state that cognitive impairment represents a common complication of type 2 diabetes mellitus (38). A meta-analysis revealed that cognitive impairment, predominantly mild cognitive impairment, affected up to 39% of patients with T2DM (35). The risk of cognitive impairment was significantly higher in the T2DM population compared with healthy individuals. Currently, over 60% of patients exhibit mild cognitive impairment, closely associated with advancing age. Its occurrence is closely associated with factors such as age, educational attainment, psychosocial status, and clinical characteristics, with individuals aged 70 and above being more susceptible to cognitive decline (39). Research demonstrates that older adults with educational attainment at secondary school level or below face a higher risk of cognitive impairment (40). Concurrently, cognitive decline is associated with elevated blood glucose levels, prolonged duration of diabetes treatment, and concomitant cardiovascular and cerebrovascular complications (41). Its pathogenesis is closely associated with reduced insulin secretion, impaired glucose homeostasis, HPA axis dysfunction, and obesity. Insulin serves not only as a peripheral metabolic regulator but also as a crucial modulator of central nervous system function. Core brain regions intimately linked to learning and memory-such as the hippocampus, prefrontal cortex, and amygdala-harbour substantial insulin receptors (42). In T2DM patients, insufficient insulin secretion inhibits central insulin signalling pathways, inducing increased neuronal apoptosis, elevated activity of *β*-secretase and *γ*-secretase, and sustained activation of glycogen synthase kinase 3β resulting in excessive phosphorylation of tau protein (43). These molecular abnormalities induce oxidative stress, activate microglia and astrocytes, and increase the release of pro-inflammatory factors such as IL-1β and TNF-*α*. This ultimately damages the function of learning and memory-related brain regions, including the hippocampus and prefrontal cortex, leading to impaired learning and memory (44).

**Figure 1:**
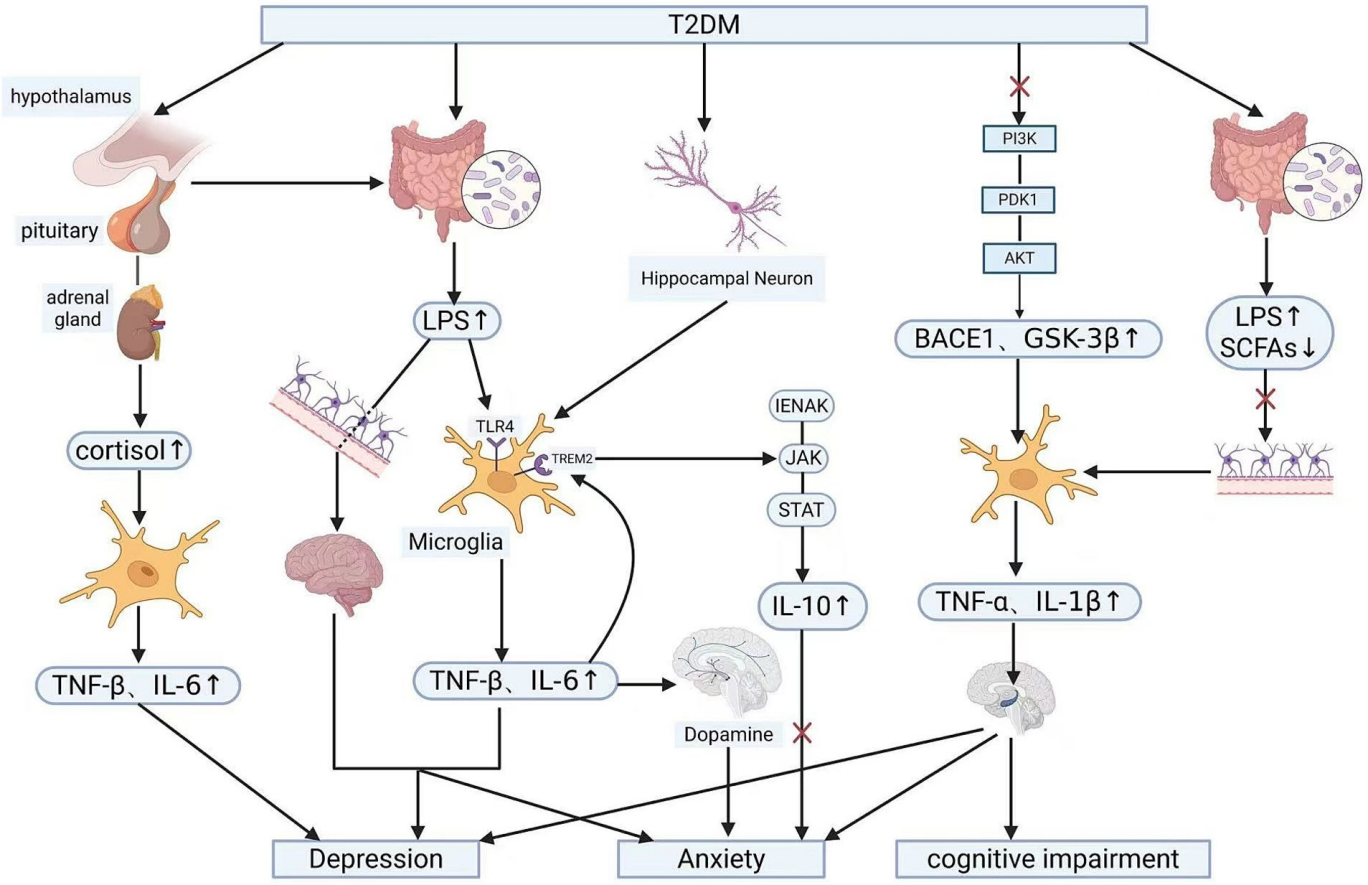
Core signalling pathways mediating the pathogenesis of psychiatric disorders induced by T2DM (created in BioRender.com). Symbol description: ×: inhibition/blockade; ↑: upregulation/elevation; ↓: downregulation/reduction. T2DM, type 2 diabetes mellitus; HPA axis, hypothalamic–pituitary–adrenal axis; Cortisol, cortisol; LPS, lipopolysaccharide; SCFAs, short-chain fatty acids; TLR4, Toll-like receptor 4; TREM2, triggering receptor expressed on myeloid cells 2; PI3K, phosphatidylinositol 3-kinase; AKT, protein kinase B; GSK-3β, glycogen synthase kinase 3β; BDNF, brain-derived neurotrophic factor; TNF-*α*, IL-1β, IL-6, pro-inflammatory cytokines.

The development of cognitive impairment is also closely linked to the gut microbiota. Dysbiosis of the gut microbiota reduces beneficial metabolites such as short-chain fatty acids while increasing harmful products (such as LPS) disrupting intestinal permeability. This allows harmful substances like LPS, TNF-*α*, and IL-6 to enter the bloodstream, subsequently damaging the blood–brain barrier (BBB). The BBB normally prevents neurotoxic compounds and pathogens from entering the brain while maintaining cerebral homeostasis. Its disruption causes dysregulation in molecular transport between the peripheral circulation and the brain, subsequently activating microglia and astrocytes to trigger neuroinflammation. This ultimately damages neuronal and synaptic function in learning and memory-related brain regions such as the hippocampus and prefrontal cortex, inducing cognitive impairment (45, 46). Moreover, these abnormalities may also precipitate psychiatric disorders such as depression and anxiety. In severe cases, T2DM patients may even develop neurodegenerative changes or dementia (47). Dementia frequently accompanies the onset of diabetes (48), and while breakthroughs have been achieved in alleviating dementia symptoms through pharmacological interventions and gut microbiota modulation, treatment protocols remain relatively incomplete. Going forward, research into the gut microbiota may yield novel therapeutic approaches to mitigate or prevent its onset and progression, thereby enhancing patient wellbeing. This figure illustrates the core molecular signalling pathways underlying the pathogenesis of psychiatric disorders, including depression, anxiety and cognitive impairment, induced byT2DM, T2DM acts as the central initiating factor, mediating the progression from metabolic disturbance to psychiatric disorders via abnormalities in the gut-brain axis, neuroinflammation, neuroendocrine function and intracellular signalling pathways (see Figure 1).

## 3 Treatment of T2DM with comorbid psychological disorders

### 3.1 Glucagon-like peptide-1 receptor agonists and dipeptidyl peptidase-4 inhibitors

Psychological distress significantly impacts the daily lives of patients with T2DM. Medication serves as the fundamental treatment approach, markedly alleviating adverse emotional states and enhancing quality of life. GLP-1 receptor agonists and dipeptidyl peptidase-4 inhibitors have become widely employed in T2DM management, representing novel therapeutic agents. Existing research has demonstrated that the therapeutic effects of GLP-1 receptor agonists such as semaglutide and liraglutide extend beyond diabetes management itself, also exerting positive influences on patients’ emotional states (49). Currently, it has been demonstrated to possess neuroprotective properties, enhance synaptic function, promote neurogenesis, and reduce oxidative stress and apoptosis (50). By activating AMPK and inhibiting the mTOR pathway, it simultaneously promotes neuronal autophagy and suppresses tau protein phosphorylation, providing a crucial molecular mechanism for ameliorating diabetes-related psychological disorders (51, 52). Furthermore, relevant studies have proposed that GLP-1 receptor agonists (GLP-1RAs) can reduce the secretion of pro-inflammatory activators by astrocytes, thereby protecting the blood–brain barrier and suppressing neuroinflammation (53).

**Figure 2:**
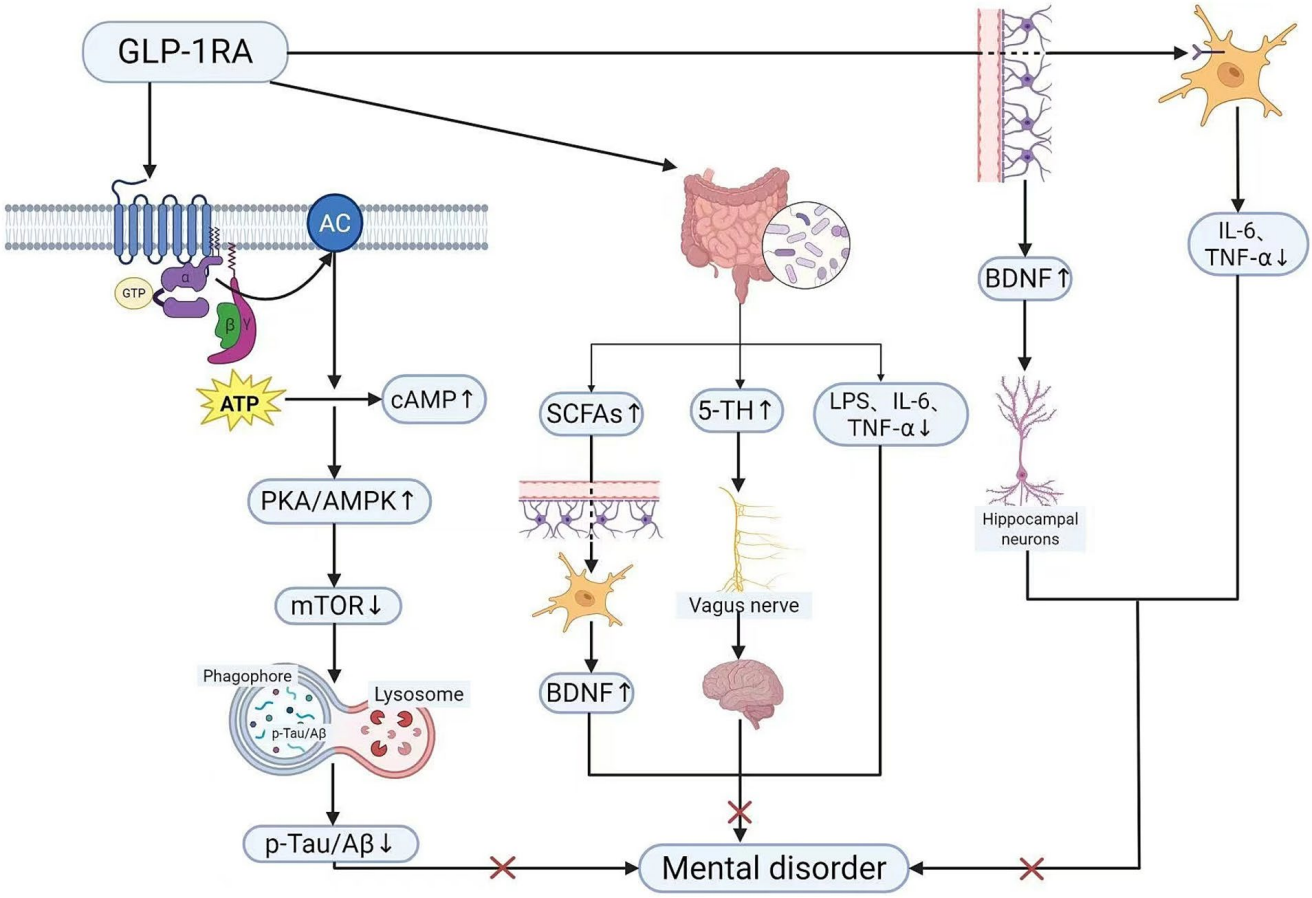
Mechanism of action of GLP-1 receptor agonists in patients (created in BioRender.com). Solid arrows: Well-established signalling pathways. Symbol legend: ×: Inhibition/blockade; ↑: Upregulation; ↓: Downregulation. GLP-1RA, glucagon-like peptide-1 receptor agonist; AC, adenylate cyclase; cAMP, cyclic adenosine monophosphate; PKA, protein kinase A; AMPK, adenosine monophosphate-activated protein kinase; mTOR, mammalian target of rapamycin; p-Tau, phosphorylated Tau protein; Aβ, β-amyloid protein; BDNF, brain-derived neurotrophic factor; NF-κB, nuclear factor kappa-light-chain-enhancer of activated B cells.

Concurrently, astrocytes release neurotrophic factors such as brain-derived neurotrophic factor, which act upon hippocampal neurons to modulate neural plasticity. This process strengthens synaptic connections between neurons, potentially alleviating psychological disorders including depression (54, 55). Of course, GLP-1 receptor agonists also exert significant effects on the gut microbiota. Glucagon-like peptide-1, produced in both enteroendocrine cells and the brain, is a hormone that regulates pancreatic function, satiety, and intestinal motility (56). It can improve the ratio of gut microbiota, increase the abundance of beneficial bacteria, and produce short-chain fatty acids (SCFAs) (57). On the one hand, SCFAs can cross the blood–brain barrier, inhibit microglial activation, promote brain-derived neurotrophic factor expression (58), thereby reducing neuroinflammation and enhancing neuroplasticity. Additionally, it stimulates serotonin synthesis in the gut, which is transported to the brain via the blood–brain barrier and vagus nerve pathways, directly ameliorating psychological states such as depression and anxiety. Concurrently, it reduces the proportion of harmful bacteria such as *Escherichia coli* and Proteus, diminishing the production of LPS and intestinal inflammatory cytokines (IL-6, TNF-*α*) (59), thereby curbing the harmful signals that trigger psychological disorders. Furthermore, relevant studies indicate that DPP-4 inhibitors such as sitagliptin and saxagliptin can also inhibit tau protein phosphorylation and promote neuronal autophagy via the AMPK/mTOR pathway (60), thereby improving cognitive dysfunction and other psychological impairments in diabetic rats. However, current research predominantly relies on animal studies. Although supported by clear mechanisms, clinical evidence in diabetic patients remains limited. Physiological disparities exist between animal studies and human trials, which may result in divergent therapeutic outcomes. Consequently, extreme caution must be exercised when translating such therapeutic interventions into routine clinical practise. Given that DPP-4 inhibitors represent a novel hypoglycaemic agent with low hypoglycaemic risk, their potential for adjunctive treatment of psychological disorders warrants further clinical investigation. This figure demonstrates the signalling pathways through which glucagon-like peptide-1 receptor agonists (GLP-1RAs) ameliorate psychiatric disorders associated with T2DM. The signalling pathways illustrated herein mainly target neuroinflammation, synaptic damage and cognitive dysfunction mediated by T2DM, exerting targeted therapeutic interventions against these pathological abnormalities (see Figure 2).

### 3.2 Metformin

Currently, metformin is the most commonly used drug for treating T2DM (61). Owing to its favourable safety profile, tolerability, and affordability, it has become the first-line treatment for T2DM (62). Clinically, the vast majority of patients take this medication for hypoglycaemic therapy. It may not only benefit medical conditions but also exert beneficial effects on core disease domains across a broad spectrum of psychiatric and neurodegenerative disorders (63). Diabetes mellitus is associated with gut microbiota dysbiosis and increased intestinal barrier permeability, allowing pro-inflammatory substances such as lipopolysaccharides to enter the bloodstream and infiltrate the central nervous system. This activates the NF-κB inflammatory pathway, triggering neuroinflammation and exacerbating psychological disorders including depression and anxiety (64, 65). Metformin activates the AMP-activated protein kinase (AMPK) pathway, directly inhibiting NF-κB activation. This reduces the release of pro-inflammatory factors such as TNF-*α* and IL-6, thereby diminishing inflammation. Consequently, it mitigates damage to hippocampal neurons and alleviates inflammation-mediated psychological and emotional abnormalities (66). After entering the mitochondrial matrix, metformin inhibits Complex I of the mitochondrial respiratory chain, thereby suppressing mitochondrial oxidative phosphorylation. This reduces ATP production, elevates the AMP/ATP ratio, and activates the AMPK pathway, producing antidepressant effects and alleviating psychological disorders (67). Also, it can directly cross the blood–brain barrier to act upon glial cells, promoting BDNF expression, enhancing neural plasticity and synaptic function, and improving antidepressant and anxiolytic-like behaviours (68). Metformin may exert effects on the gut microbiota by increasing the abundance of beneficial bacterial groups such as Akkermansia and Proteobacteria, strengthening the intestinal mucus barrier, and repairing the intestinal barrier. This reduces levels of LPS entering the bloodstream and decreases pro-inflammatory factors like IL-6, thereby mitigating neuroinflammation and alleviating damage to hippocampal neurons. Consequently, it exerts beneficial effects on patients with psychological disorders associated with diabetes (69). According to the latest data analysis, metformin use was associated with a significantly reduced risk of depression (70). Animal studies have demonstrated that metformin significantly reduces levels of 3-nitrotyrosine in the rat hippocampus, lowers pro-inflammatory factors such as TNF-*α* and IL-6, and increases the content of the anti-inflammatory factor IL-10, thereby mitigating neuronal damage. This provides experimental evidence for its indirect amelioration of psychological disorders (71). Nevertheless, these findings are still derived from preclinical animal studies. While they demonstrate the anti-inflammatory potential of metformin, the clinical significance of its association with 3-NT in patients with diabetes mellitus remains unclear. Furthermore, conclusions drawn from animal experimentation cannot be fully extrapolated to human subjects. Accordingly, the relevance of this biomarker for clinical application warrants further investigation.

**Figure 3:**
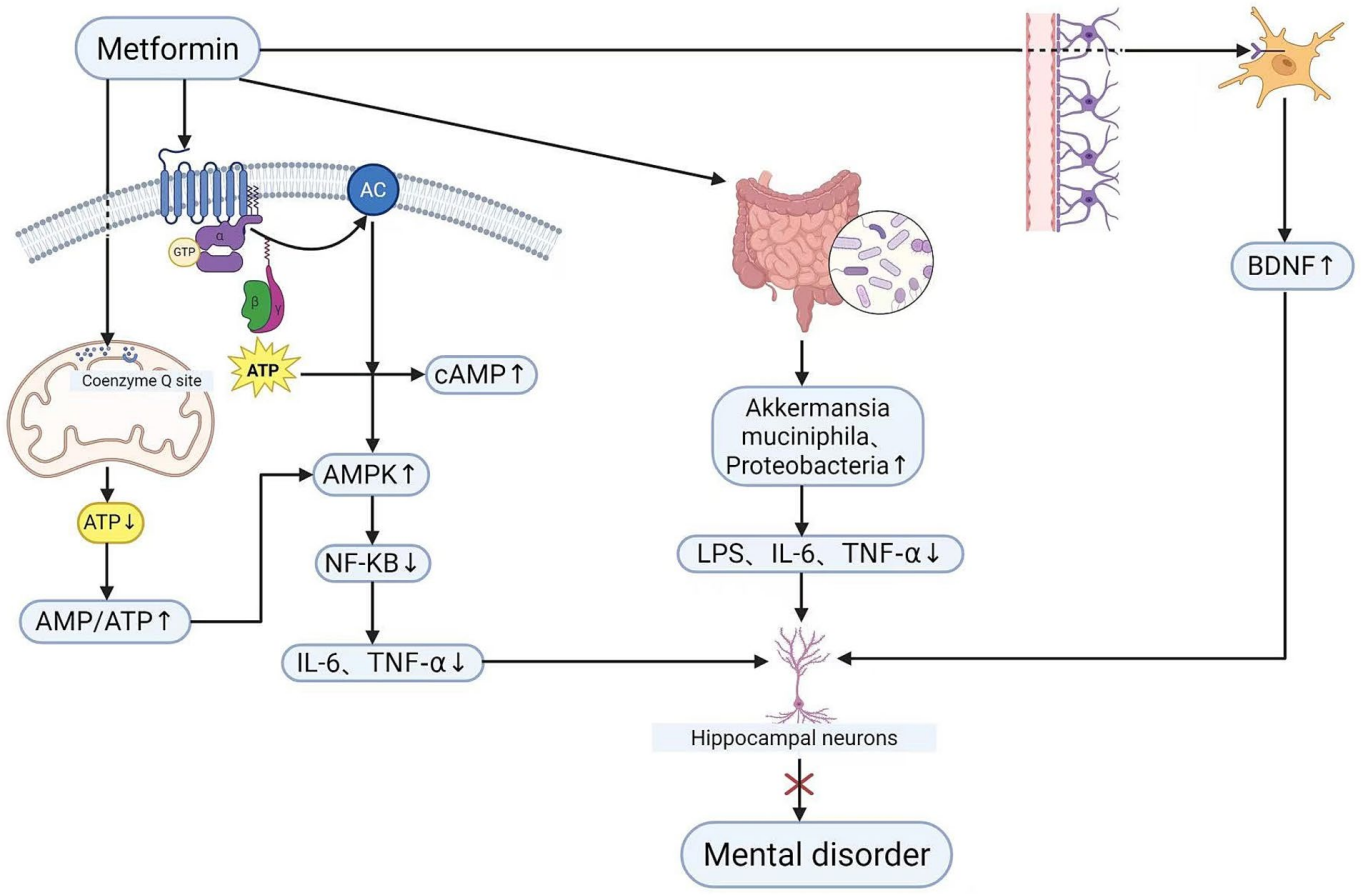
Mechanism of action of metformin in patients (created in BioRender.com). Solid arrows: Well-established signalling pathways. Symbol legend: ×: Inhibition/blockade; ↑: Upregulation; ↓: Downregulation. Metformin, metformin; AMPK, adenosine monophosphate-activated protein kinase; NF-κB, nuclear factor kappa-light-chain-enhancer of activated B cells; TNF-α, IL-6, pro-inflammatory cytokines; LPS, lipopolysaccharide; SCFAs, short-chain fatty acids; BDNF, brain-derived neurotrophic factor.

Current research has demonstrated a molecular association between nitric oxide and the PI3K/AKT pathway, which serves as a central pathway for cellular responses to external stimuli. Metformin can influence cellular metabolism, inflammatory responses, and homeostasis by modulating the activity of this pathway. Concurrently, it can mitigate neuronal damage and improve energy metabolism in nerve cells by regulating this pathway, thereby offering potential benefits for central nervous system disorders (72). However, current research on psychological disorders associated with diabetes has not explored the relationship between the PI3K/AKT pathway and reactive nitrogen species accumulation. Going forward, clinical practise could involve detecting changes in this pathway and reactive nitrogen species accumulation through targeted regulation. By subsequently analysing depression scale scores alongside alterations in synaptic plasticity molecules and inflammatory factors, the underlying mechanisms may be elucidated. This approach could offer novel insights for future clinical practitioners. This figure illustrates the protective mechanisms of metformin against psychiatric disorders associated with T2DM, confirms that metformin ameliorates T2DM-induced neuroinflammation and neuronal damage via both AMPK-dependent and AMPK-independent signalling pathways (see Figure 3).

Beyond the aforementioned medications, a promising novel therapeutic approach for diabetes currently focuses on modulating the gut microbiota through probiotics, prebiotics, and faecal microbiota transplantation (73). Modulating the microbiome can influence metabolism and mental health. Probiotics and prebiotics such as inulin-type fructans and dietary fibre can restore gut microbial balance, enhance intestinal barrier integrity, and promote the growth of beneficial bacteria. This facilitates the reduction of endotoxinaemia and stabilises patients’ emotional states. Concurrently, they may stimulate the release of beneficial gut hormones and microbial metabolites, thereby improving glycaemic fluctuations and insulin resistance in T2DM patients, exerting significant positive effects (74, 75). Relevant animal studies indicate that ginsenoside Rh4 and ginsenoside compound K can mitigate damage to hippocampal neurons and synaptic structures by suppressing immune inflammatory responses and signalling molecule interaction pathways (28, 76), thereby reducing excessive pro-inflammatory cytokine expression and glial cell overactivation. Furthermore, they improve gut dysbiosis, significantly enriching beneficial bacteria such as Akkermansia and increasing short-chain fatty acid content, which alleviates depressive and cognitive impairment behaviours in mouse models. As potential gut microbiota modulators, these findings provide novel theoretical support for addressing psychological issues in diabetic patients. Although these results demonstrate certain beneficial effects in animal experiments, the clinical efficacy of ginsenosides in patients with diabetes mellitus has not yet been fully validated. Similar to the aforementioned animal studies, this conclusion remains restricted to preclinical animal models, and the scarcity of human studies means that it is not possible to confirm whether such effects can be replicated in clinical settings. In addition, alterations in gut microbiota may exhibit substantial interindividual variability, which may also compromise the generalisability of the experimental findings.

### 3.3 Psychological, dietary and exercise interventions

In addressing psychological distress amongst T2DM patients, treatment extends beyond medication to encompass lifestyle modifications that effectively and conveniently alleviate negative emotions. Healthcare professionals may employ psychological, dietary and exercise interventions to enhance quality of life. Psychological interventions for diabetic patients help alleviate negative emotions, reduce the development of psychological disorders, and improve both glycaemic control and quality of life (77). Cognitive behavioural therapy (CBT) and structured psychological therapy are particularly relevant at present. As a non-pharmacological intervention, CBT is widely used in the psychological management of patients with diabetes mellitus. Its primary aim is to modify negative cognitions and behaviours and reduce psychological distress including anxiety and depression. A meta-analysis reported that CBT significantly reduces glycated haemoglobin and fasting blood glucose levels, while also improving symptoms of depression and anxiety (78). It enhances coping with diabetes-related stress and improves self-management, thereby supporting glycaemic control.

In addition, structured psychological therapies, especially supportive and problem-solving approaches-have demonstrated beneficial effects in this population by addressing practical challenges in disease management and alleviating diabetes-related psychological burden (79). Dietary interventions also offer a potential avenue for preventing or mitigating depression, anxiety, and diabetic symptoms, effectively lowering the risk of psychological disorders (80). Evidence suggests that the Mediterranean diet—comprising vegetables, whole grains, fruit, lean protein, and healthy fats-can improve blood glucose fluctuations and reduce cardiovascular risk in patients (81), while also alleviating psychological distress and mitigating negative emotions (82). Enhancing dietary composition with fruits, vegetables, and dairy products may prevent mental disorders by modulating the gut microbiota (83).

Research demonstrates that high-fibre diets alter gut microbiota, increasing the abundance of beneficial bacteria such as Lactobacillus, Bifidobacterium, and Akkermansia, while reducing the abundance of Clostridium, Klebsiella, and other opportunistic pathogens. This subsequently improves patients’ blood glucose homeostasis, serum metabolism, and emotional fluctuations (64). For instance, the Dietary Approaches to Stop Hypertension diet, owing to its high dietary fibre content (84), promotes the production of short-chain fatty acids by gut microbiota (85). This facilitates postprandial glucose reduction and enhanced insulin sensitivity (86), conferring significant protective effects.

**Figure 4:**
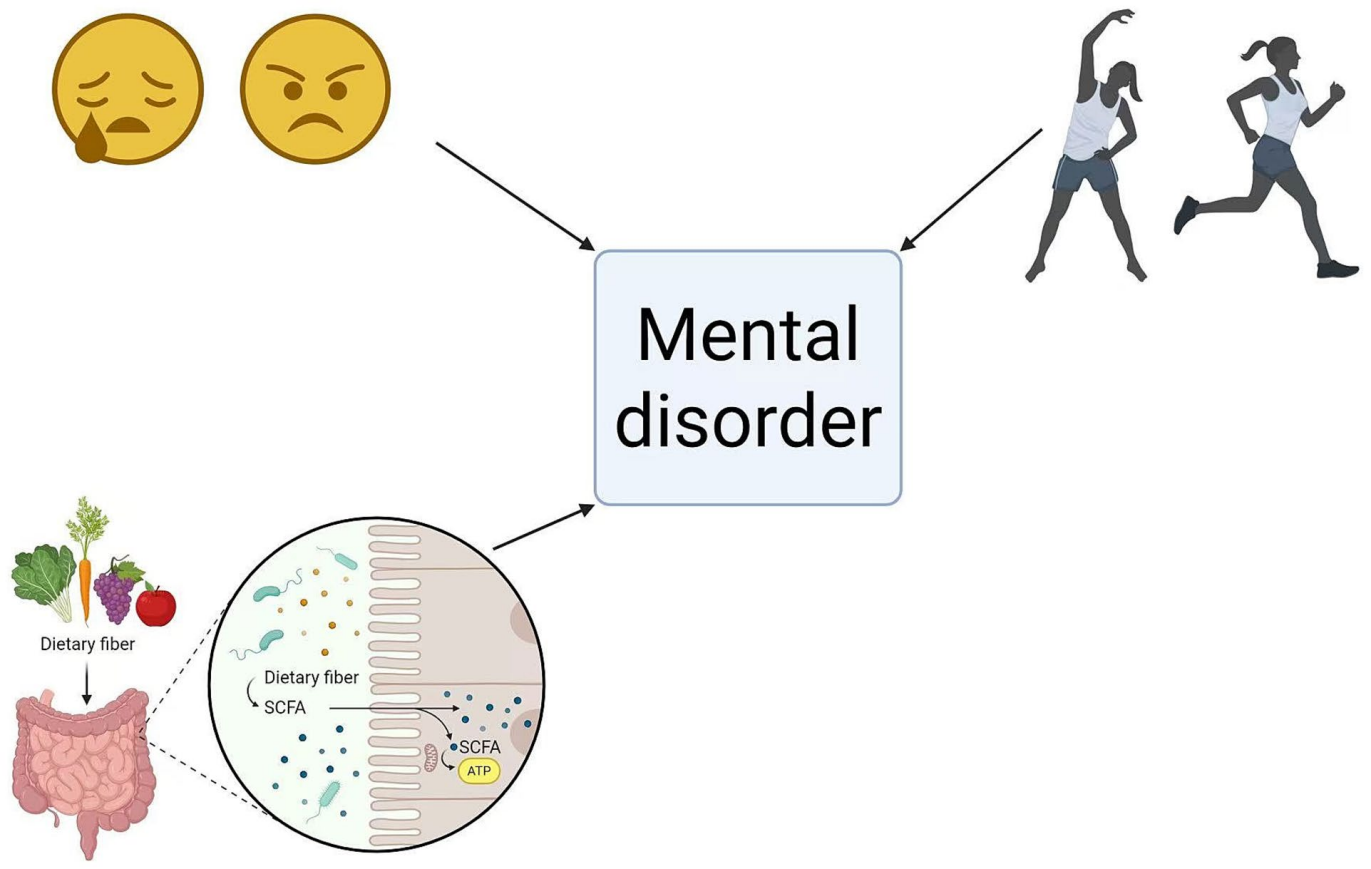
Effects of psychological, dietary, and exercise therapies on patients (created in BioRender.com). Solid arrows: Well-documented and validated signalling pathways. SCFAs, short-chain fatty acids; BDNF, brain-derived neurotrophic factor; LPS, lipopolysaccharide.

Concurrently, moderate exercise benefits patients’ physical and mental wellbeing while alleviating life pressures. Aerobic exercise (AE) ameliorates adverse emotional states (87). Research indicates AE effectively mitigates depressive and anxious symptoms through multiple mechanisms, including modulation of neuroinflammation, cerebral neuroplasticity, growth factor expression, the hypothalamic–pituitary–adrenal axis, and the microbiota-gut-brain axis, thereby promoting holistic patient health (88). For instance, activities such as swimming (89), moderate-intensity treadmill exercise (89), Bagua Zhang (90), and yoga (91)can improve glycated haemoglobin levels. These not only enhance insulin sensitivity (92), but also reduce cerebral oxidative stress, lower pro-inflammatory factor levels, and improve patients’ psychological state, thereby fostering positive emotional responses. This suite of interventions can assist patients in alleviating adverse psychological states such as anxiety and depression, thereby enhancing their confidence in treatment outcomes. This figure depicts the signalling pathways whereby lifestyle factors, encompassing diet, physical activity and emotional state, exert regulatory effects on psychiatric disorders associated with T2DM via intestinal metabolism. The pathways illustrated herein highlight the critical value of the gut-metabolism-brain axis as a non-pharmacological target for the intervention and management of T2DM complicated with comorbid psychiatric disorders (see Figure 4; Table 1).

**Table 1:** Summary of clinical interventions for neuropsychological disorders.

Interventions	Mechanism of action	Level of evidence	Current clinical status
GLP-1RA	1. Activates AMPK and inhibits the mTOR pathway, promoting neuronal autophagy and suppressing Tau protein phosphorylation. 2. Reduces the secretion of pro-inflammatory activators by astrocytes. 3. Upregulates BDNF in astrocytes and modulates cerebral neural plasticity. 4. Acts on the gut microbiota: on the one hand, increases short-chain fatty acids (SCFAs), which cross the blood–brain barrier and enhance BDNF expression; on the other hand, promotes intestinal serotonin synthesis and decreases the production of lipopolysaccharide (LPS) and intestinal inflammatory cytokines including IL-6 and TNF-α.	Level 3 (moderate-quality cohort studies + small-sample randomised controlled trials)	Existing clinical evidence demonstrates that such agents may serve as adjunctive therapy to improve cognitive scores and alleviate affective disorders in patients with diabetes mellitus and comorbid psychological disorders. Several large prospective cohort studies and small-sample randomised controlled trials have confirmed their potential modulatory effects on tau-protein-related pathological processes. However, high-quality evidence from large-scale long-term follow-up studies remains insufficient for primary neurodegenerative disorders and severe cognitive impairment.
Mechanism	1. Activates the adenosine monophosphate-activated protein kinase (AMPK) pathway, suppresses the activation of nuclear factor-kappa B (NF-κB), and reduces the release of pro-inflammatory cytokines such as tumour necrosis factor-alpha (TNF-α) and interleukin-6 (IL-6). 2. Enters the mitochondrial matrix, inhibits Complex I of the mitochondrial respiratory chain, suppresses mitochondrial oxidative phosphorylation, thereby reducing adenosine triphosphate (ATP) production and elevating the AMP/ATP ratio, which in turn activates the AMPK pathway. 3. Can penetrate the blood–brain barrier (BBB) directly to act on neuroglial cells and upregulate the expression of brain-derived neurotrophic factor (BDNF). 4. Akkermansia acts on the gut microbiota to increase the abundance of beneficial bacteria including and Proteobacteria, strengthens the intestinal mucus layer barrier, lowers the level of circulating lipopolysaccharide (LPS), and reduces pro-inflammatory cytokines such as IL-6.	Grade2b (multiple moderate-quality cohort studies plus small-sample randomised controlled trials, leading to clinical consensus)	Its adjunctive applications in the field of mental disorders have gained widespread clinical consensus. Several moderate-quality clinical studies have confirmed that it can slow the rate of cognitive decline in patients with diabetes mellitus and ameliorate comorbid mood disorders. However, as a monotherapy for primary neuropsychological disorders in the absence of metabolic abnormalities, its efficacy is limited, and combination with other therapeutic interventions is generally required.
Lifestyle	1. Psychological therapy: cognitive behavioural therapy (CBT) and structured psychotherapy. 2. Dietary therapy: including the Mediterranean diet, high-fibre diet, Dietary Approaches to Stop Hypertension (DASH), modulation of the gut microbiota, and increased short-chain fatty acid production. 3. Exercise therapy: aerobic exercise (AE) modulates neuroinflammation, cerebral neuroplasticity, growth factor expression, the hypothalamic–pituitary-adrenal (HPA) axis, and the microbiota-gut-brain axis.	Grade 1a (top-level evidence from multiple high-quality meta-analyses, endorsed by clinical guidelines)	Lifestyle intervention represents a first-line intervention for the management of mental disorders. Its limitations include a high dependence on patient adherence for therapeutic efficacy, the absence of standardised quantitative protocols, limited effectiveness when used as monotherapy in patients with moderate to severe neuropsychological disorders and cognitive disorders, and the requirement for concomitant pharmacotherapy. Furthermore, it exhibits a relatively slow onset of action in the short term, with sustained clinical benefits only observable following long-term implementation. As such, it is suitable as a fundamental, whole-course intervention integrated throughout the phases of diagnosis, treatment and rehabilitation.

## 4 Conclusion

T2DM is a chronic metabolic disorder requiring lifelong glycaemic control. Due to the protracted treatment duration, patients are prone to developing psychological issues such as depression, anxiety, and cognitive impairment. These conditions are closely associated with factors including age, gender, lifestyle, social support capacity, psychosomatic stress, complications, medication exposure, obesity, dietary patterns, socioeconomic status and inflammatory responses. This review examines the pathogenesis of depression, anxiety, and cognitive impairment, alongside pharmacological treatments and psychological, dietary, and exercise interventions for these psychological issues. It further elucidates their close association with the gut microbiota, where the microbiota-gut-brain axis plays a significant role. While existing research suggests that hypoglycaemic agents may exert beneficial effects in the mitigation of psychiatric disorders, the majority of current studies investigating DPP-4 inhibitors have been conducted predominantly in preclinical animal models, with comparatively limited data derived from clinical investigations. Notably, there is a marked scarcity of clinical trial data evaluating the impact of DPP-4 inhibitors on psychiatric outcomes, and this evidence gap currently restricts the broader clinical application of these agents in patients diagnosed with psychiatric disorders. Moving forward, there is a clear need for the design and implementation of multicentre, large-scale randomised controlled trials (RCTs) to assess the specific therapeutic effects of DPP-4 inhibitors on psychiatric comorbidities in patients with diabetes mellitus. To enhance the generalisability of research findings, such clinical trials ought to enrol diverse patient populations encompassing varying ethnicities, age groups, durations of diabetes, and those with concurrent comorbidities. In parallel, substantial scope remains for further mechanistic exploration of the MGBA pathway; future research efforts should aim to elucidate the causal linkages mediated by this pathway between diabetes mellitus and the pathogenesis of psychiatric disorders. Such mechanistic investigations will serve to provide robust theoretical support and empirical clinical evidence to inform and optimise targeted therapeutic strategies in clinical practise.

## 5 Prospects

### 5.1 Effects of SGLT-2 inhibitors in T2DM patients comorbid with psychological disorders

Sodium-glucose cotransporter 2 (SGLT-2) inhibitors, including dapagliflozin and empagliflozin, play a pivotal role in the long-term glycaemic control of patients with type 2 diabetes mellitus (T2DM). In recent years, however, a growing body of research has shifted focus to their neuropsychiatric implications. Emerging neuropsychiatric evidence suggests that SGLT-2 inhibitors may exert favourable effects on the mental health of diabetic patients, with studies documenting improvements in diabetes-related depressive and anxiety symptoms (93). Nevertheless, current research into the neuropsychiatric effects of this drug class remains limited.

To elucidate the precise role of SGLT-2 inhibitors in psychiatric comorbidities amongst diabetic populations, future investigations should prioritise multicentre, large-sample RCTs coupled with long-term longitudinal follow-up. Such studies are warranted to explore the sustained benefits of these agents across emotional disorders, cognitive function and health-related quality of life. Furthermore, given the interindividual variability amongst T2DM patients, personalised therapeutic strategies and multidisciplinary collaboration should form a core focus of subsequent research. These approaches will enable a comprehensive assessment of the neuropsychiatric profile of SGLT-2 inhibitors, supporting the development of holistic and optimised treatment regimens for T2DM patients with concurrent psychological disturbances.

### 5.2 Psychopharmacological therapy and its interplay with glycaemic management

Psychotropic medications, notably antidepressants, anxiolytics and antipsychotics, are widely prescribed in T2DM patients with comorbid psychological disorders. However, a complex bidirectional interaction exists between psychopharmacological agents and glycaemic control. Several classes of antidepressants, particularly selective serotonin reuptake inhibitors (SSRIs), have been associated with adverse metabolic effects including weight gain, increased appetite and insulin resistance, which may exacerbate glycaemic dysregulation and complicate diabetes management (94). Conversely, select antidepressants such as fluoxetine have been shown to alleviate diabetes-associated anxiety and depressive symptoms to a certain extent, thereby indirectly facilitating better glycaemic control (95).

Despite existing preliminary research, current understanding of the interplay between psychotropic medications and diabetes remains incomplete. Future studies must adopt a multidimensional framework to evaluate the metabolic and psychiatric impacts of these agents, encompassing mechanistic investigations of distinct drug classes, drug–drug interactions, and the design of individualised treatment protocols. Leveraging multidisciplinary teamwork, large-scale clinical trials and advanced technological methodologies will support the delivery of targeted, personalised psychopharmacological care, ultimately optimising both glycaemic control and quality of life in T2DM patients with psychiatric comorbidities.
